# The role of pallidotomy in the precision medicine era

**DOI:** 10.3389/fneur.2026.1735969

**Published:** 2026-02-13

**Authors:** Giacomo Garone, Alice Innocenti, Alessandro De Benedictis, Maria Camilla Rossi-Espagnet, Franco Randi, Donatella Lettori, Simone Reali, Flaminia Frascarelli, Alessandra Savioli, Silvia Cossu, Laura Cantonetti, Nazaret Infante, Nicola Specchio, Carlo Efisio Marras

**Affiliations:** 1 Neurology, Epilepsy and Movement Disorders Unit, Bambino Gesù Children's Hospital, IRCCS, Rome, Italy; 2 Neurosurgery Unit, Bambino Gesù Children's Hospital, IRCCS, Rome, Italy; 3 Diagnostic and Interventional Neuroradiology Unit, Bambino Gesù Children's Hospital, IRCCS, Rome, Italy; 4 Unit of Neurorehabilitation, Bambino Gesù Children's Hospital, IRCCS, Rome, Italy; 5 Anesthesia and Critical Care Unit, Bambino Gesù Children's Hospital, IRCCS, Rome, Italy; 6 Neurology Unit, Pediatric Hospital Antonio Cao, Brotzu Hospital Trust, Cagliari, Italy; 7 Department of Neurosurgery, Hospital del Mar, Barcelona, Spain; 8 Epilepsy Neuroscience Research Group, Hospital del Mar Research Institute, Barcelona, Spain; 9 University Hospitals KU Leuven, Leuven, Belgium; 10 Systems Neurology and Neurotherapeutics Lab Neurosciences Program, Hospital del Mar Research Institute, Barcelona, Spain

**Keywords:** ablative surgery, children, dystonia, pallidotomy, radiofrequency

## Abstract

**Background:**

The use of radiofrequency pallidotomy (RP) for medically refractory dystonia has markedly declined since the introduction of deep brain stimulation (DBS). Conditions such as severe cognitive impairment, poor nutritional status, or acquired dystonia—common in children with severe dystonia—may limit DBS eligibility or benefit. In these cases, RP may represent a valuable alternative. Evidence on RP in this population remains scarce, mostly based on small case-series with limited follow-up. We retrospectively analyzed RP safety, feasibility and long-term outcomes in children with medically refractory dystonia.

**Methods:**

Records of patients who underwent RP at Bambino Gesù Children’s Hospital were reviewed. Outcomes were retrospectively assessed through the Clinical Global Impression-Improvement scale. For patients with a history of status dystonicus (SD), recurrence was used as outcome measure.

**Results:**

Eighteen patients underwent 21 procedures. Sixteen received bilateral simultaneous pallidotomy; two unilateral pallidotomy after GPi-DBS removal due to infection. Three patients required repeated surgery for recurrent-SD. Ten had acquired dystonia (cerebral palsy, CP), five genetically confirmed dystonia, three idiopathic dystonia. Three were followed for three months or less; in the others, mean follow-up was 6.62 ± 3.65 years. Three months postoperatively, 12 of 16 patients improved (1 “very much improved,” 2 “much improved,” 9 “minimally improved”), 2 were unchanged and 2 minimally worsened. At last follow-up (15 patients), 1 remained “very much improved,” 2 “minimally improved,” 3 “unchanged,” 6“minimally worsened,” 3 “much worsened.” Among six patients treated for ongoing-SD, crises abated over 50.7 ± 27.8 days. SD recurred in 8 patients (5 CP, 1 genetic, 2 idiopathic) after a mean of 20.3 ± 19.8 months.

**Conclusion:**

RP emerges as a feasible rescue-therapy for severe medically refractory SD. Most patients show meaningful short-term improvement, but beneficial effects tend to decline over time, with high rate of SD recurrence—particularly in dyskinetic-CP. This may reflect suboptimal lesions’ location and/or size, progressive disease course or re-emergence of dystonia through different networks. In this light, RP should be considered when DBS is contraindicated, the risk of hardware-related complications outweighs the advantages of an adjustable system, or in patients with limited life expectancy, where an irreversible yet effective rescue intervention is clinically justified.

## 1 Introduction

Radiofrequency pallidotomy (RP), consisting of radiofrequency lesioning of the internal globus pallidus (GPi), was first performed in 1947 and soon after became widely adopted to treat Parkinson’s disease (PD) (1). After the advent of L-DOPA in the 1960s, the use of ablative surgery for movement disorders gradually diminished up until the 1980s and early 1990s, when it resurged as an effective treatment for motor symptoms of advanced stage PD. At that time, RP emerged as a potentially effective treatment also for dystonia, as newer models of basal ganglia function identified the GPi and the substantia nigra pars reticulata as the main output stations of the basal ganglia network (2, 3). Consequently, suppressing abnormal pallidal output in dystonia was considered a potentially effective strategy to achieve better control of motor symptoms. RP application decreased substantially once again after the advent of DBS, that largely replaced ablative surgery (1, 4). Since the effective introduction of pallidal DBS for dystonia at the beginning of the 21st century, RP has been gradually reserved for patients with limited DBS applicability (5).

Factors limiting DBS eligibility in dystonic patients may include severe cognitive impairment, cachectic state, predictable failure to comply with follow-up and hardware-related costs (4). These concerns are commonly encountered in children with severe, medically refractory dystonia, in which DBS-related complications are more common compared to adults (6). Additionally, both acquired dystonia and status dystonicus (SD) are more common in children than in adults, both representing possible reasons to opt for ablative surgery, given the limited efficacy of DBS in acquired dystonia and the potentially life-threatening risk of SD.

A recent review of individual patient data undergoing bilateral RP for dystonia highlights its efficacy in the short term, but the number of studies reporting long term outcomes of individuals undergoing RP is limited and the assessment is heterogenous (4).

Because of the limited available evidence, the role of bilateral RP in severe pediatric dystonia is still debated (5).

In this study, we conducted a retrospective analysis of a pediatric cohort of medically refractory dystonic children and young people who underwent RP at our center, in order to evaluate safety, feasibility and long-term outcomes.

## 2 Participants and methods

### 2.1 Charts review and patients’ selection

Medical charts of children and young people with acquired, genetic or idiopathic dystonia who underwent RP at the Bambino Gesù Children’s Hospital between 2011 and 2023 have been retrospectively reviewed using a structured proforma. Clinical data were retrieved from their medical files and video recordings, when available.

Patients with disabling dystonia refractory to medical treatment were considered eligible to surgical treatment. Medically refractory dystonia was defined after failed attempts of treatment with ≥2 medications or in case of failure to abate SD after appropriate medical management, in accordance with ongoing expert recommendations (7). According to expert recommendations (8), the factors underpinning the decision to opt for RP instead of DBS included: (i) high risk of hardware-related complications because of extreme dystonic posturing, poor nutritional status, small body dimensions; (ii) life-threatening, medically refractory SD requiring urgent treatment and preventing time-consuming programming sessions; (iii) severe pre-existing neurological deficits including severe speech and feeding disturbances, which mitigate the concerns for bilateral RP-induced side effects; (iv) limited willingness or feasibility of attending the programming center of the patient and their caregiver; and (v) previous complications from an implanted DBS system, when the severity of the condition precluded the possibility of waiting for the time required to perform a new implantation.

Some of the patients included in this study have already been described in previous publications from our group (9, 10).

### 2.2 Preoperative assessment

As part of the preoperative work-up, to visualize basal ganglia and vascular structures, patients underwent brain MRI with 3D T1-, T2-, inversion recovery and gadolinium-enhanced T1-weighted images. A preoperative volumetric CT scan was performed in all patients. A full video-taped neurological examination was performed before the surgery, except in cases where the severity of the clinical conditions, the urgency of the procedure, or the state of sedation in the intensive care unit did not allow for a meaningful or feasible recording.

### 2.3 Surgical procedure

All surgeries were performed under general anesthesia, through a frameless stereotactic neuronavigation robotic system (ROSA ONE^®^ Brain, Zimmer Biomet, Warsaw, IN, United States).

Over the study period, anesthesia protocols changed and were progressively adapted according to evolving clinical practice. In general, anesthesia induction was achieved using one or more hypnotic agents (propofol, midazolam, and/or sevoflurane) combined with an opioid analgesic (fentanyl or sufentanil). Anesthesia maintenance was achieved with intravenous anesthesia using propofol or midazolam as hypnotic agents and remifentanil or morphine as analgesics. When available, continuous monitoring of anesthesia depth was performed using bispectral index (BIS™) monitoring (Medtronic plc, Dublin, Ireland).

The target was localized to the mid portion of the GPi, and was preoperatively identified by visual direct targeting through volumetric inversion recovery, T2, FLAIR and post-contrast T1-weighted brain MRI sequences merged with brain CT.

Intraoperative neurophysiological monitoring was individualized and protocols varied during the study period. Microelectrode recordings (MER) and/or macrostimulation under surface EMG and/or flash visual evoked potentials (fVEPs) and Transcranial Electric Motor Evoked Potentials (tcMEPs) were performed (Leadpoint MER acquisition System – Medtronic, Inc.; Cascade Elite and Iomax systems – Cadwell Industries, Inc.), to detect the nucleus and to avoid the involvement of relevant surrounding structures by the lesioning (i.e., internal capsule and optic tract), respectively.

MER recording was performed along the planned trajectory from 10 mm above to the target.

The ablation was performed through a radiofrequency generator (Cosman RFG-1; Cosman Medical Inc., Burlington, MA, United States) on the target and along a trajectory of 8 mm above the target, at 40 V for 60 s. A second and a third trajectory (2 mm posterior, 2 mm lateral to the first target) was used to perform lesioning. The standard entry point for the procedure was located in close proximity to the coronal suture, approximately 20 mm lateral to the midline. This trajectory allows the exploration of the nucleus along the final 10 mm of the trajectory, before entering the target. Alternative trajectories (e.g., a more lateral entry point or a transventricular approach) were used in patients with distorted brain anatomy, resulting from severe cerebral atrophy and/or ventricular enlargement.

### 2.4 Postoperative assessment and outcome measures

All patients performed brain imaging (CT scan or MRI) within 24 h from surgery. The decision to perform CT or MRI was guided by the patient’s clinical status, immediate availability, the clinical suspicion of intraoperative complications and the possible presence of a DBS system contralateral to the lesion. For patients who underwent immediate CT scan, brain MRI was postponed to the following days. Late brain MRI (≥30 days after surgery) was performed according to clinical judgement, based on the patients’ clinical status.

No standardized protocol for postoperative clinical evaluation was implemented, in line with the aim of minimizing logistical burdens and patient discomfort in this frail cohort.

As outcome measure after surgery, the Clinical Global Impression–Improvement scale (CGI-I) was retrospectively rated by two child neurologists based on medical records, integrating caregivers’ impressions of improvement or worsening, findings from neurological examinations, and changes in medical treatment, including medication escalation or de-escalation. In patients treated for ongoing SD, resolution was defined as the discontinuation of intravenous sedative therapy, and the interval between surgery and SD resolution was calculated accordingly. For patients with previous episodes of SD, recurrence of further SD episodes was annotated and employed as an outcome measure. Recurrent SD was defined as the occurrence of a new episode of dystonic exacerbation requiring admission to an intensive care or high-dependency unit for intravenous sedative treatment.

## 3 Results

### 3.1 Study cohort

During the study period, 18 patients underwent 21 surgical procedures. Sixteen patients performed bilateral simultaneous RP. Two patients, who had been previously implanted with GPi-DBS and in which one of the DBS electrodes was removed because of a hardware infection, underwent unilateral RP. In both cases, RP was performed using a standard surgical technique after removal of the infected electrode during a previous procedure. In one patient, the contralateral DBS system was left in place. In the other patient, the DBS system had been explanted bilaterally; however, RP was performed unilaterally because the contralateral GPi had been previously damaged by a perielectrodal intracranial hemorrhage at the time of DBS implantation, rendering it an unsuitable target for RP. In both patients, ablative surgery was chosen over DBS reimplantation because conservative management of the hardware infection failed to avoid DBS explanation, and delayed DBS reimplantation was considered unsuitable given the severity of the underlying dystonia and previous SD episodes.

Three patients with previous bilateral surgery underwent a second RP during the follow up period due to SD recurrence. Ten patients had acquired dystonia (Dyskinetic cerebral palsy), 5 patients had definite genetic dystonia, and 3 patients had dystonia of unknown etiology (but likely genetic) (Table 1).

**Table 1:** Clinical features of study participants.

Pt. ID	Etiology	Age range at 1° RP (years)	Indication	Timing	Side	Time to SD resolution (days)^a^	Time to SD relapse (days)^a^	Advanced medical devices/interventions before surgery	Age range at 2nd RP (years)/indication	Total follow-up duration (years)
Pt. 01	CP	15–20	Previous SD	Semi-urgent	Bilateral	NA	670,00	PEG, ITB	20–25/medically refractory SD	8.95^b^
Pt. 02	Isolated generalized dystonia of unknown cause	20–25	Severe dystonia	Elective	Left	NA	/	DBS	NA	0.24
Pt. 03	CP	15–20	Severe dystonia	Elective	Bilateral	NA	/	PEG	NA	0.1
Pt. 04	CP	15–20	Severe dystonia	Elective	Bilateral	NA	11,00	NGT feeding	NA	0.05
Pt. 05	Encephalopathy with epilepsy and generalized dystonia, unknown cause	15–20	Medically refractory SD	Urgent	Bilateral	55,00	932,00	PEG, tracheostomy	NA	13.50
Pt. 06	*GNAO1*-related movement disorder	15–20	Previous SD	Elective	Left	NA	NA	DBS	NA	4.18
Pt. 07	CP	<5	Previous SD	Elective	Bilateral	NA	NA	/	NA	0.37
Pt. 08	*TUBB4A*-related hypomyelination with atrophy of the basal ganglia	<5	Previous SD	Semi-urgent	Bilateral	NA	NA	PEJ	NA	2.85
Pt. 09	CP	10–15	Previous SD	Semi-urgent	Bilateral	NA	310,00	PEG, ITB	NA	7.36
Pt. 10	CP	5–10	Severe dystonia	Elective	Bilateral	NA	NA	/	NA	2.96
Pt. 11	DEE (multigene deletion chromosome 22p)	10–15	Medically refractory SD	Urgent	Bilateral	20,00	NA	PEG, tracheostomy	NA	5.04
Pt. 12	*GNAO1*-related movement disorder	10–15	Previous SD	Semi-urgent	Bilateral	NA	79,00	PEG, tracheostomy	NA	2.93
Pt. 13	*NEXMIF*-related Intellectual developmental disorder	<5	Medically refractory SD	Urgent	Bilateral	27,00	NA	PEG	NA	3.72
Pt. 14	CP	<5	Previous SD	Semi-urgent	Bilateral	NA	NA	/	NA	9.04
Pt. 15	Generalized dystonia with bilateral striatal necrosis, unknown cause	<5	Medically refractory SD	Urgent	Bilateral	33,00	1729,00	PEG	10–15/medically refractory SD	5.12^b^
Pt. 16	CP	10–15	Medically refractory SD	Urgent	Bilateral	100,00	NA	ITB	NA	11.78
Pt. 17	CP	10–15	Medically refractory SD	Urgent	Bilateral	70,00	141,00	PEG, tracheostomy	NA	12.35
Pt. 18	CP	5–10	Medically refractory SD	Urgent	Bilateral	50,00	1,008,00	PEG	10–15/previous SD	5.15^b^

CP, cerebral palsy; DEE, developmental and epileptic encephalopathy; RP, radiofrequency pallidotomy; SD, status dystonicus; NA, not applicable; PEG, percutaneous endoscopic gastrostomy; PEJ, percutaneous endoscopic jejunostomy; NGT, nasogastric tube; ITB, intrathecal baclofen pump; DBS, deep brain stimulation.

a From the date of the RP procedure.

b From first RP to last follow-up post second RP.

At the time of surgery, 12 patients were receiving enteral feeding through gastrostomy, jejunostomy or nasogastric tube, 4 had a tracheostomy for respiratory failure and 3 were already on intrathecal baclofen pump treatment.

### 3.2 Surgery

Mean age at first surgery was 11.97 ± 5.87 years. Indication for surgery included: urgent intervention for ongoing, medically refractory status dystonicus (7 patients); semi-urgent surgery for previous medically managed SD with residual severe dystonia requiring priority surgery to avoid SD recurrence (7 patients); and elective surgery for severe medically refractory dystonia without previous SD (4 patients). In the three patients undergoing a second pallidotomy, mean age at reintervention for second lesion surgery was 15.23 years (range 10.1–23.5 years), with a mean interval between surgeries of 4.5 years (range 3.3–5.3 years). In two of them, second-step RP was performed during an ongoing, medication-refractory SD. The third patient was operated after the resolution of a previous, medically managed SD. Mean time between surgeries was 4,52 years (range 3.34–5.33 years).

During anesthesia, BIS™ monitoring was recorded and documented in 13 out of 21 surgeries. During anesthesia maintenance and surgery (including intraoperative recordings) BIS was maintained between 35 and 50.

In 14 procedures, MER was performed to detect GPi activity and improve target localization. In 9 patients, a spiking pattern compatible with GPi activity (i.e., high activity, rapidly firing neurons) was documented within the targeted area. In the remaining 5 cases, GPi electrical activity was not detectable or doubtful. Notably, in all of these 5 patients, preoperative MRI showed pallidal signal abnormalities. Motor evoked potentials were performed on 10 patients, and visual evoked potentials on 8 patients.

### 3.3 Follow-up and post-surgical outcomes

**Figure 1:**
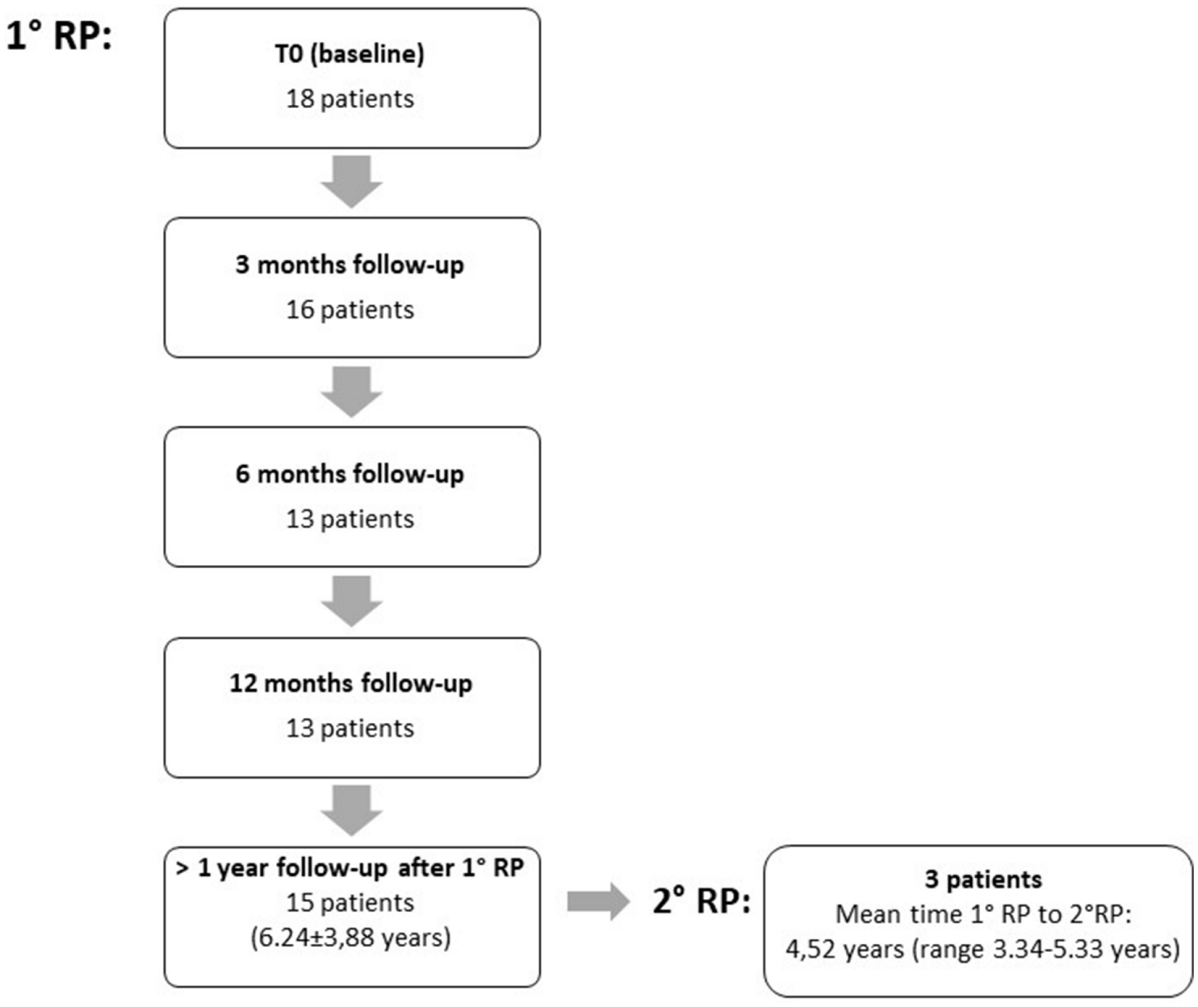
Number of patients at each follow-up time point.

Three patients had been referred for surgery from other Institutions and were monitored only for the immediate post-operative period (i.e., equal or less than 3 months). The mean follow-up time, for the other 14 patients, was 6.62 ± 3.65 years, with two patients missing the scheduled follow-up assessments at 6 and 12 months. In total, 16 patients were evaluated at 3 months post-surgery, 13 at 6 months and 12 months and 15 had a last follow-up more than 1 year after surgery (Figure 1). During the follow-up period, two patients died for medical complications unrelated to pallidotomy.

#### 3.3.1 Status dystonicus management

For the seven patients who underwent urgent pallidotomy for medically refractory SD, the median time from SD onset to surgery was 56.14 days (range 13 to 139 days). SD abated over a mean time of 50.71 ± 27.81 days (range 20–100 days) after surgery. However, in the days preceding SD resolution, patients required less intensive treatment, reduced sedative therapy and were successfully transferred from the ICU to the general ward.

#### 3.3.2 Short-term post-surgical outcomes

**Figure 2:**
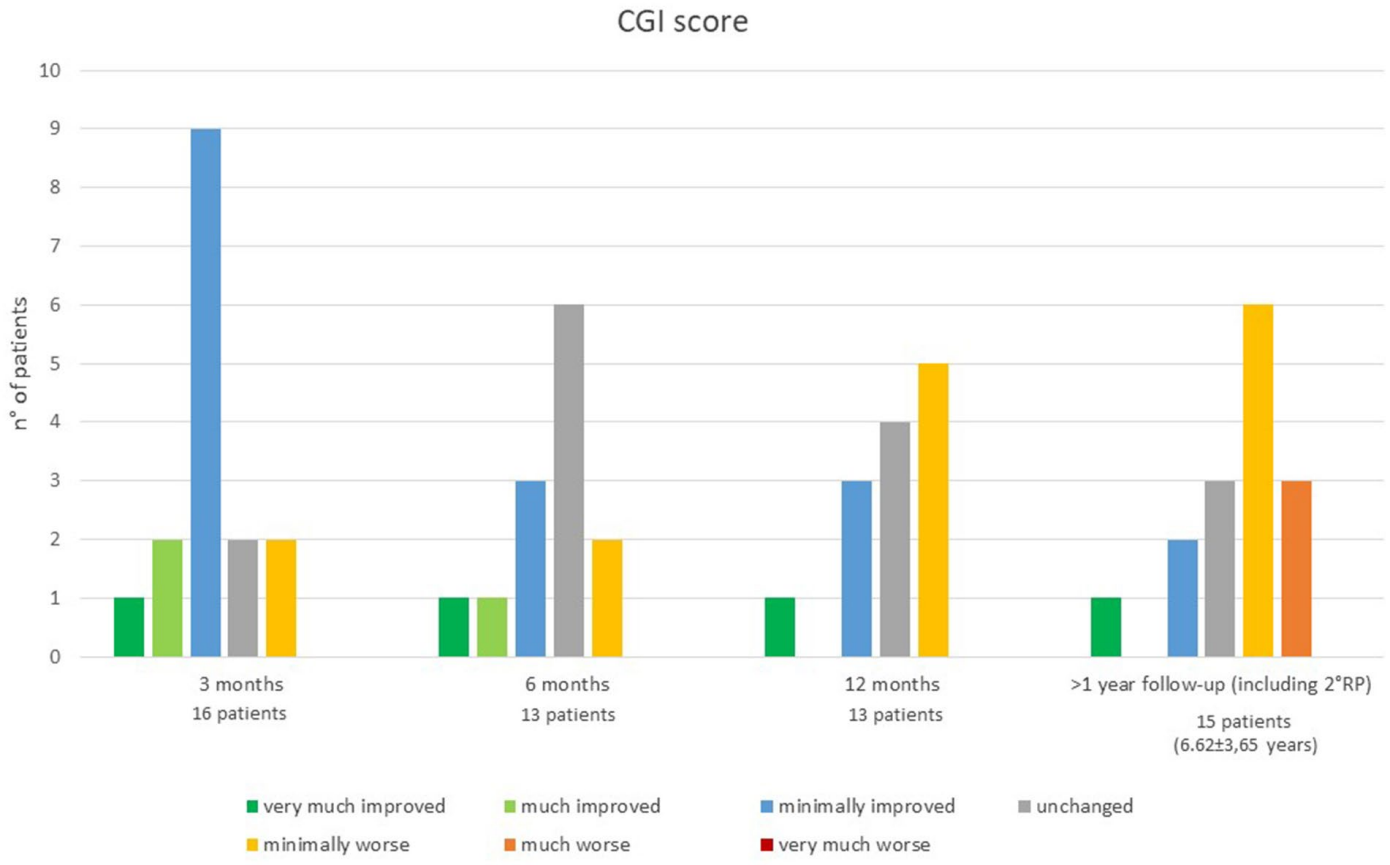
Clinical Global Impression scale at each follow-up time point.

Three months after surgery, 12 out of 16 patients showed a global improvement of dystonia (described as “very much improved in 1,” “much improved” in 2 and “minimally improved” in 9), 2 patients were described as unchanged and 2 patients as minimally worsened. At 6 months, out of 13 patients, 1was described as “very much improved,”1 as “much improved” and 3 as “minimally improved,” while 6 patients were “unchanged” and 2 were “minimally worsened” compared to their baseline (two patients missed the scheduled assessment) (Figure 2).

#### 3.3.3 Long-term post-surgical outcomes

One year after surgery, out of 13 patients, 4 still reported some improvement (1 “very much improved”; 3 “minimally improved”), 4 patients were reported as unchanged respect to their baseline and 5 patients as minimally worsened. At last follow-up, out of 15 patients, compared to baseline 1 patient was still described as “very much improved,” 2 patients as “minimally improved,” 3 as “unchanged,” 7 as “minimally worsened,” and 3as much “worsened” (Figure 2).

#### 3.3.4 Re-pallidotomy outcomes

Out of the three patients undergoing second-step pallidotomy, one did not recover from SD and died during ICU hospitalization. In a second patient, SD abated within 10 days, but starting from the 6th postoperative month, gradual worsening of dystonia was reported. The third patient (operated after a previous medically managed SD), minimal improvement was reported in the 6 months following the second procedure, with subsequent dystonia worsening.

### 3.4 Post-operative neuroimaging

**Figure 3:**
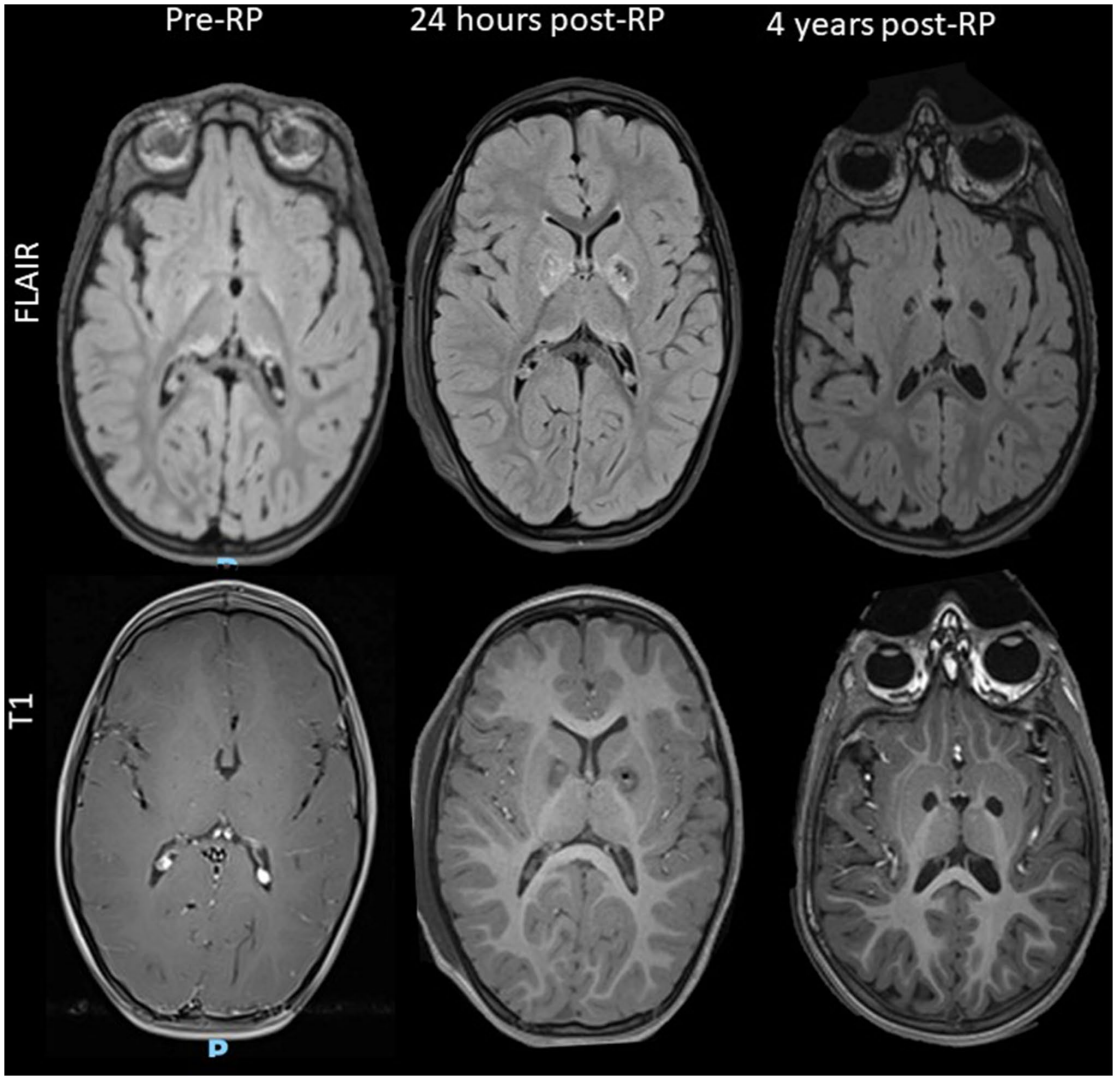
Evolution of MRI imaging after RP in a representative patient (subject #7). Preoperative MRI shows subtle bilateral FLAIR hyperintensities in the globi pallidi and thalami. Early postoperative imaging demonstrates a necrotic core with surrounding diffuse oedema involving the striata and internal capsule, while late MRI depicts cavitating evolution of the lesion with oedema resolution.

**Figure 4:**
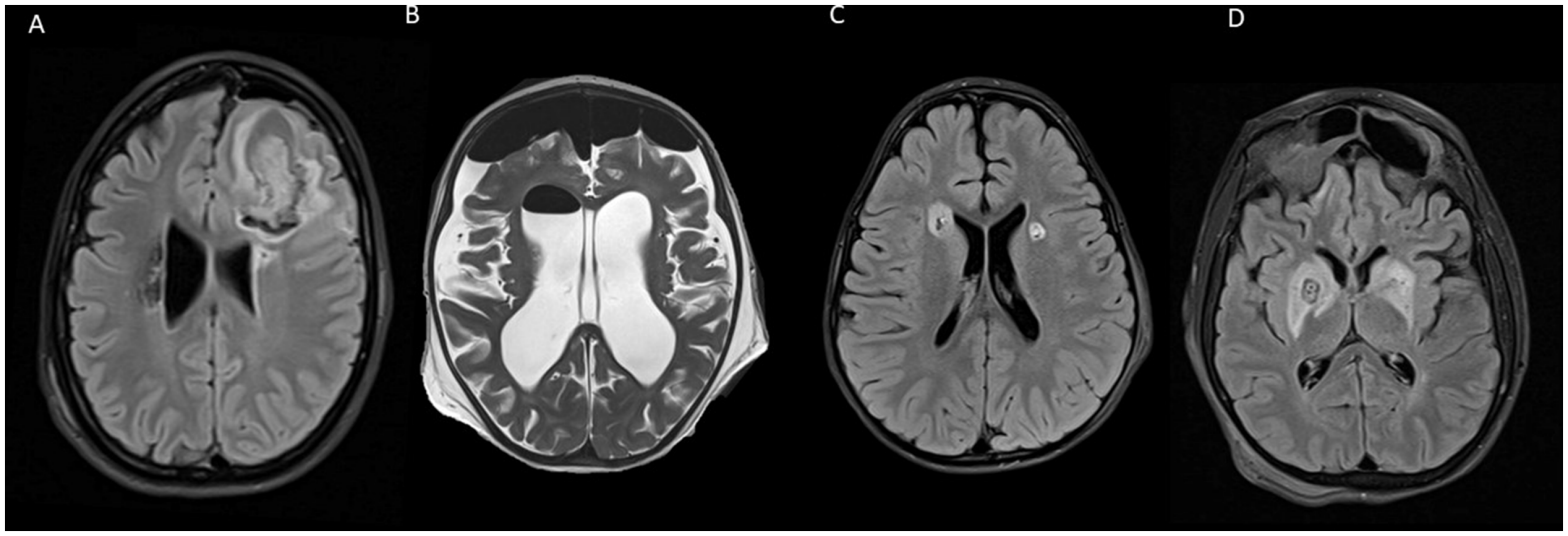
Radiologic complications after RP. **(A)** Frontal bleeding along the surgical trajectory. The patient presented with contralateral hemiplegia, motor apraxia, and cognitive decline. **(B)** Pneumoventricle and frontal pneumoencephalon. **(C)** White matter FLAIR hyperintensity along the surgical trajectory. **(D)** Diffuse oedema involving the striatal nuclei bilaterally.

In the first post-operative day, MRI was performed in 8 surgeries and CT scan in 13. For patients whose MRI was postponed, the exam was performed 13.33 ± 10.11 days later (range 2–34 days). One patient did not perform postoperative MRI due to the presence of a contralateral DBS system. Two out of three patients undergoing double pallidotomy did not perform any MRI scan after the second surgery. Early MRI (within the first 2 postoperative days) displayed limited hemorrhagic foci within the lesion, in all subjects. In 6 out of 8 patients performing MRI within 30 days after surgery, a limited hemorrhagic focus within the lesion was still evident (Figure 3). In addition to intralesional bleeding, one patient experienced a frontal intraparenchymal hemorrhage with a slight midline shift, two patients had asymptomatic subdural bleeds, and one patient developed a bilateral frontal pneumocephalus with unilateral pneumoventricle (Figure 4). In 14 surgeries, the first postoperative imaging detected extrapallidal extension of the lesion or perilesional oedema; involving the putamen (6 cases), internal capsule (3 cases) or both (3 cases).

In 8 cases, a second MRI was performed after a variable time delay (37.25 ± 44.39 months, range 1–139 days). In 5 cases, extrapallidal extension of the necrotic lesion was detected: to the putamen (1 case), internal capsule (2 cases), both (1 case) or in the white matter along the surgical trajectory (1 case).

**Figure 5:**
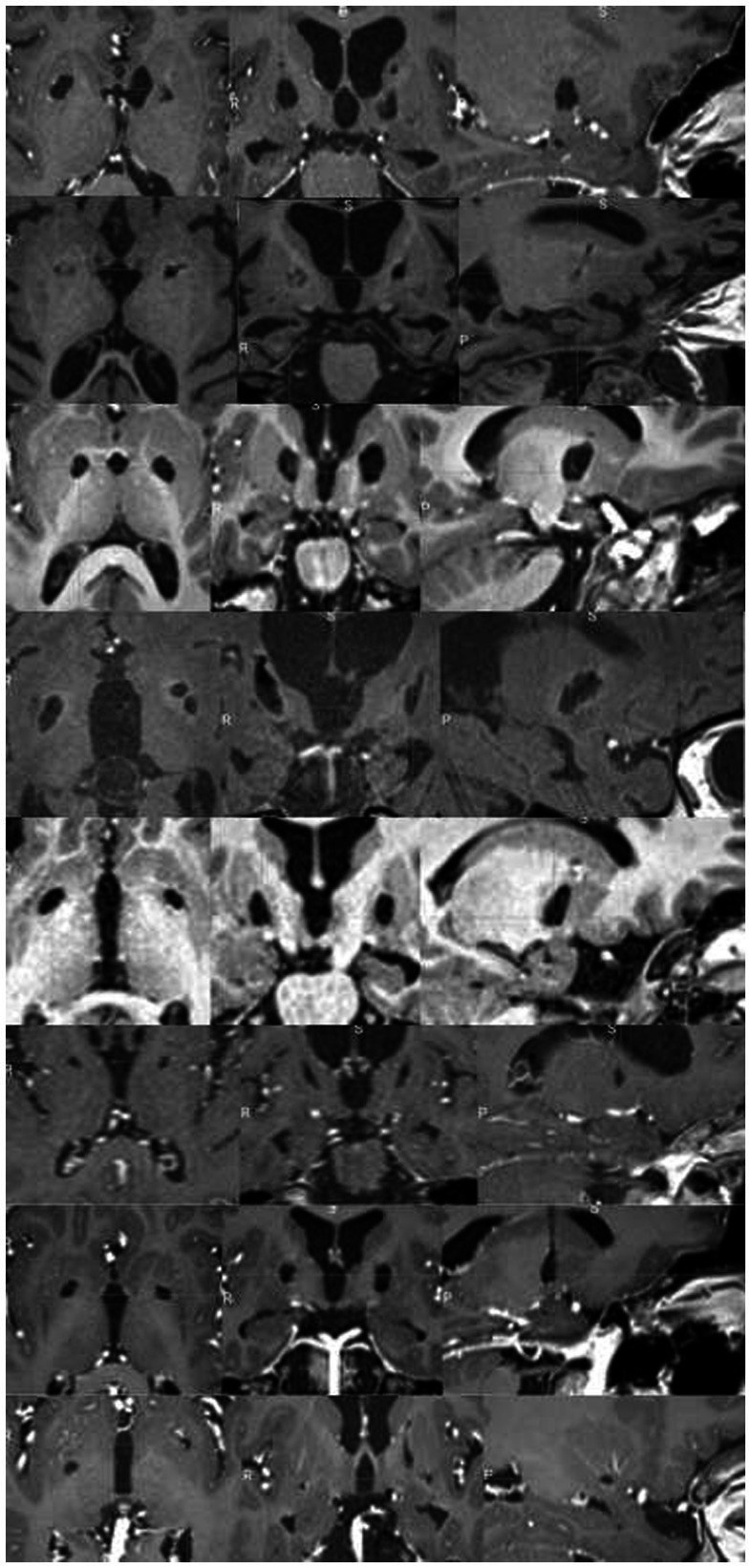
Chronic evolution of GPi lesions in subject undergoing late MRI scans. Each rows represent a single patient T1-weighted MRI sequence on axial, coronal, and sagittal view (from up to bottom: patient #1, #5, #7, #8, #14, #15, #17, #18). In all patients, bilateral lesion was still visible.

In all patients undergoing late MRI, RP lesion was still visible on both sides (Figure 5), including in the three patients who underwent re-pallidotomy for lesion extension.

### 3.5 Adverse events

Overall, adverse events were reported after 9 surgeries, 3 of which were procedure-related, while 6 were due to medical complications. The adverse events related to the surgical procedure included: motor apraxia, cognitive decline and behavioral changes succeeding frontal hemorrhage in 1 patient, and hemiplegia associated with global akinesia in 2 other patients. Adverse events related to the post-surgical management included: respiratory distress (3 patients, 2 of which required tracheostomy due to extubation failure), post-surgical sepsis (2 patients), ventilator associated pneumonia (1 patient) and deep vein thrombosis (1 patient).

## 4 Discussion

Given the rise of DBS popularity and its effective application in managing severe pediatric dystonia, the role of ablative surgery remains uncertain (5). In the past years, some authors argued that pallidotomy had been prematurely abandoned, citing the overall low rate of adverse events and the good rate of improvement (11). Nevertheless, evidence for safety and efficacy of pallidotomy in dystonia (especially in children) mostly relies on case reports or small case series with limited follow up time (4), limiting evidence quality and comparability with DBS.

DBS is preferred over pallidotomy as treatment for severe dystonia because of its reversibility, the adjustability of stimulation (which makes treatment adaptable to evolving clinical scenarios) (4) and the possibility of performing bilateral surgery with a lower rate of postoperative neurological deficits compared to ablative surgery (especially in terms of postoperative dysarthria and dysphonia). On the other hand, hardware-related complications are a major source of morbidity in patients with severe dystonia [especially in children, Koy (6)], making the risk-to-benefit ratio less evidently weighted in favor of DBS in children with expected limited response to DBS, increased risk of hardware-related complications, pre-existing severe neurological deficits and/or life-threatening emergencies (such as SD) (8). In such cases, a RP targeting the middle third of the GPi may be strategic to avoid precluding a future DBS implantation in the posteroventral GPi, if required.

The aim of this study is to describe the safety and efficacy of pallidotomy in a cohort of children with severe pediatric dystonia and limited DBS applicability, with long-term follow up for most of the patients.

### 4.1 RP in status dystonicus

From our cohort, RP emerges as an effective therapy for medication-refractory SD and severe dystonia. All patients undergoing the first surgery for medication-refractory SD were successfully discharged from ICU. The time needed for SD resolution after surgery was highly heterogenous, with many patients requiring weeks of intravenous sedative treatment after surgery. Nevertheless, RP facilitated the tapering of sedative therapy and enabled successful transfer from the ICU to lower-intensity care units. These findings are in line with a previous report suggesting that RP is effective for SD in various forms of dystonia (12).

In two patients with relapsing medication-refractory SD after a previous successful management of SD by RP, a second surgery to extend the first lesion was effective to abate a SD only in one patient.

### 4.2 Short term outcomes

A positive effect on dystonia was found in the first months after surgery in most patients, regardless of the indication for surgery. These findings suggest that, in the short term, RP is effective in relieving dystonic symptoms. The therapeutic effects were not immediately visible but took several days to weeks to become detectable; some patients suffered transient exacerbations of dystonia in the early postoperative course.

### 4.3 Long term outcomes

After the first 6 months after surgery, an overall trend toward dystonia reemergence was observed. Similarly, SD recurred after RP in a significant proportion of patients with previous SD episodes (7 out of 14 patients). This observation is in line with previous reports of long-term follow up after unilateral RP for hemidystonia (13), where some patients were found to worsen over time. Several possible, non-mutually exclusive explanations may account for the long-term loss of therapeutic effect and increased risk for SD in a significant portion of the patients. First, it is likely that a progressive disease course contributed to dystonia worsening in some patients with an underlying neurodegenerative condition. Nevertheless, a gradual loss of improvement was observed also in patients with static diseases, such as dyskinetic cerebral palsy, with 5 out of 10 patients with acquired dystonia experiencing SD relapses after pallidotomy. An inadequately placed or sized lesion is a second possible explanation, as distorted brain anatomy in this population often compromises GPi targeting by reducing imaging contrast and landmark reliability and increasing susceptibility to brain shift. Inconsistency in postoperative imaging timing prevent us from a formal analysis of lesion size and location. Nevertheless, in all the patients performing late postoperative scans lesion was still clearly visible on MRI, including patients with SD relapse. This finding suggests that dystonia reemergence is not due to lesion shrinkage, differently from what has been observed for tremor after MRI-guided focused ultrasound (MRIgFUS) thalamotomy (14).

Another contributing mechanism could be the reemergence of dystonia through alternative networks, such as the cerebello-thalamic network. This hypothesis is supported by multiple lines of evidence suggesting that dystonia may arise from the dysfunction of a broad network including the motor and somatosensory cortices, the cerebellum, thalamus and basal ganglia (15), enabling network reorganization after ablative surgery. A similar hypothesis has been argued to explain the reemergence of Parkinson’s Disease-related tremor after MRIgFUS thalamotomy. In this case, a possible propagation of tremor to a pallidothalamic network has been postulated (16). In the three patients from our cohort who underwent lesion extension through second-stage pallidotomy, surgery was less effective compared to first-stage surgery, possibly supporting the hypothesis of a shift in the underlying dystonia network organization. Notably, in a subset of patients with gradual worsening of dystonia after transient improvement, intraoperative MER had failed to identify pallidal neuronal activity. While this could reflect deep anesthesia or suboptimal localization, the concomitant presence of pallidal MRI hyperintensities may indicate markedly reduced neuronal activity, suggesting that preserved GPi functional integrity could influence RP outcome. Accordingly, preoperative multimodal assessment with 18F-FDG PET and structural/functional MRI connectivity may help predict surgical outcome (17, 18).

Regardless of the mechanisms of dystonia recurrence after surgery, the individual outcome remains difficult to predict. Therefore, patients and families should be informed that the initial surgical benefit may diminish over time. In our cohort, no clear-cut differences in terms of outcome were observed based on dystonia etiology (genetic, idiopathic or CP), possibly due to the limited size of our sample and the lack of a standardized post-operative assessment.

Moreover, advances in the understanding of genetic basis of dystonia and the identification of genetic predictors of DBS response have prompted a re-evaluation of the traditional exclusion criteria for neuromodulation. For instance, GNAO1-related dystonia is now known to respond dramatically to DBS (14), and, in the present day, it would be considered first line invasive treatment, even in fragile patients. Unfortunately, many patients with genetic dystonia in our cohort underwent surgery prior to the completion of their diagnosis, or before data on DBS responsiveness of their specific condition became available. This is particularly relevant for patients with (yet) undetermined dystonia at the time of surgical evaluation. When genetic diagnosis is unknown, DBS should not be excluded solely based on the severity of their clinical picture, especially in absence of overt brain abnormalities on MRI.

### 4.4 Adverse events

Although the overall rate of postoperative adverse events was substantial, the majority were medical complications, likely reflecting prolonged hospitalization and the generally poor clinical condition of the patients. Small intralesional hemorrhages and perilesional edema extending into surrounding structures were frequently observed on early postoperative scans. These may reflect an increased risk of lesion expansion from thermal dispersion, pneumocephalus, hemorrhage, and brain shift in patients with complex or distorted brain anatomy, particularly when transventricular trajectories are employed. Nevertheless, adverse events directly related to surgery only occurred in 3 out of 21 patients and were generally mild. One patient experienced cognitive decline and motor apraxia after frontal hemorrhage, whereas two patients experienced hemiplegia and hypokinesia that improved over time and did not significantly impact their functional status. Considering that most of the patients had previous neurological deficits with absent speech and severe feeding difficulties, no overt worsening of dysarthria and dysphonia was reported. However, in two patients requiring postoperative tracheostomy, worsening of the bulbar function, with increased drooling and ineffective airway clearance, may have contributed to extubation failure.

### 4.5 Limitations

Our results should be interpreted in light of some limitations. We did not perform any standardized assessment of dystonia severity in the follow up; our retrospective evaluation of treatment outcome may suffer from reporting, observer and loss-at-follow up biases. Our cohort was highly heterogenous in terms of dystonia etiology and indication for surgery. Furthermore, only a subgroup of our cohort performed a tardive postoperative brain MRI, impeding evaluation of exact size and location of the lesions and their correlation with clinical outcome.

## 5 Conclusion

In conclusion, RP emerges as a potentially effective rescue therapy for severe dystonia and medically refractory SD, especially in life-threatening clinical situations. Most of the patients show clinically meaningful improvement in the first months after surgery. Nevertheless, beneficial effects on dystonia appear to diminish in the long term, and the risk of recurrent SD is elevated. Given these considerations, RP should be considered as treatment for severe dystonia and SD when DBS is contraindicated, when the risk of hardware-related complications overcomes the advantages of an adjustable treatment, and in patients with limited life expectancy. MRI-guided approaches, such as focused ultrasound or laser interstitial thermal therapy, may extend the therapeutic applications of pallidotomy by ensuring greater lesioning precision and, in the case of focused ultrasound, incisionless surgery. Such techniques are increasingly used to treat movement disorders in adults (19–21), and have been occasionally applied in children (22). Improved procedural accuracy is expected to lead to better outcomes and lower rates of adverse events, thereby enhancing the clinical applicability of these approaches, particularly in patients with severe disability and frail health. Nevertheless, access to these techniques—as well as to DBS—remains limited by their high costs, leaving room for RP in many countries, where it may represent the only therapeutic option.

## Data Availability

The data analyzed in this study is subject to the following licenses/restrictions: since original data of this study consist of medical records, they are not publicly available due to privacy restrictions. The data that support the findings of this study are available on request from the corresponding author. Requests to access these datasets should be directed to AI, alice.innocenti@opbg.net.

## Author’s note

GG and AI Full Member of European Reference Network EpiCARE.
